# Impact on Road Safety and Operation of Rerouting Traffic in Rural Travel Time Information System

**DOI:** 10.3390/s20154145

**Published:** 2020-07-25

**Authors:** Mariusz Kiec, Carmelo D’Agostino, Sylwia Pazdan

**Affiliations:** 1 Faculty of Civil Engineering, Cracow University of Technology, Warszawska 24, 31-155 Cracow, Poland; mkiec@pk.edu.pl (M.K.); sylwia.pazdan@pk.edu.pl (S.P.); 2 Transport & Roads Division, Faculty of Engineering, Lund University, John Ericssons väg 1, Box 118, 221 00 Lund, Sweden

**Keywords:** ITS, road safety, travel time information system, safety performance function

## Abstract

The Travel Time Information System (TTIS) is an Intelligent Traffic Control System installed in Poland. As is common, travel time is the only factor in the decision about rerouting traffic, while a route recommendation may consider multiple criteria, including road safety. The aim of the paper is to analyze the safety level of the entire road network when traffic is rerouted on paths with different road categories, intersection types, road environments, and densities of access points. Furthermore, a comparison between traffic operation and road safety performance was carried out, considering travel time and delay, and we predicted the number of crashes for each possible route. The results of the present study allow for maximizing safety or traffic operation characteristics, providing an effective tool in the management of the rural road system. The paper provides a methodology that can be transferred to other TTISs for real-time management of the road network.

## 1. Introduction

The Travel Time Information System (TTIS) was implemented in Poland in 2012 with the aim to solve the problem of seasonal congestion of the road network in the recreation area of the Malopolska region. This complex Intelligent Transport System (ITS) covers both national and regional rural and suburban roads. The aim of the TTIS is the effective exploitation of capacity reserves existing in the road network by providing information to road users about alternative routes with a lower traffic density and shorter travel time. 

The traffic redistribution on the road network not only impacts the environment (fuel consumption and an increase in pollution) but it can affect in a not negligible way the safety of the road users. The common problem in the implementation of this kind of complex system is that the travel time (measured as traffic volume in relation to the infrastructure capacity) is the only factor in the decision about rerouting traffic instead of considering multiple criteria [1]. The present research aims to analyze the effects of the TTIS system on road safety and travel time by verifying the overall safety and traffic operation performance of the road network, including national and regional roads and their intersections where traffic is rerouted. The road safety assessment was carried out by calibrating Safety Performance Functions (SPFs) for national and regional roads (with a greater density of access points), for both sections and intersections. The use of SPFs calibrated on the basis of empirical data offered the advantage to predict the safety conditions of the whole road network included in the TTIS for different values of rerouted traffic. Furthermore, by assessing the safety level of each road section and intersection as a function of the Annual Average Daily Traffic (AADT) and geometric parameters, it was possible to simulate in real time the network performance in terms of road safety. This was possible since the effects of the TTIS system did not change the risk related to the different road categories and intersection types (the estimated crash modification factor for the TTIS was close to 1, as shown in [2]). In fact, by rerouting traffic, the TTIS affects crash frequency in non-homogenous road categories and intersection designs. A comparison of the traffic operation and road safety was also assessed, looking for the optimum of two measures of system operation, changes in crashes and travel time.

This paper is a further step to develop a preliminary analysis on the safety performance of the network when traffic is rerouted between paths with different road segment categories and characteristics [2]. The mentioned study has high reliability in the methodology and data but was conducted only in terms of road safety for road sections, while intersections were not included in the analysis.

## 2. Literature Review

The TTIS, recommending alternative routes on the basis of travel time, is commonly used. It improves the level of service and may have controversial effects on road safety on the main regional and national road networks based on the magnitude of the rerouted traffic [2]. This preliminary study [2] considers the effects of the system by developing simulated scenarios of traffic redistribution on the network. The main problem of those applications is that the TTIS may also influence the change in traffic at intersections. It may lead to a greater number of crashes than those related only to road sections, based on the different risks related to the specific crossed intersections [3].

The added value of including intersections in the overall analysis of the real-time safety conditions of the network is due to the fact that drivers may more frequently change their travel routes, preferring routes with low priority and a shorter travel time. This may result in a greater number of dangerous maneuvers at intersections and consequently a greater probability of multiple vehicle road crashes.

The TTIS is more often implemented in urban areas than on rural roads. For example, such a system operates in the Norwegian city of Trondheim [3] and Hong Kong [4]. A similar experience was carried out in London, and showed an increase in the number of crashes in connection with the increase in the proportion of vehicles equipped with connected on-board tools for rerouting [4]. Assuming a total share of vehicles equipped with on-board tools to be 100%, the costs of road crashes will increase by 1.5% [5]. Other studies suggest that the distribution of traffic in the suburban road network, relying on the shortest travel time while still maintaining an acceptable level of service, led to an increase in the risk of crashes (considering a non-linear relationship between crashes and traffic volume) [6,7]. In all of those systems, the basic principle is to reroute traffic in the road network to minimize the delays (travel time) of users. Based on the real-time traffic volume, the system calculates the traffic performance and gives information about alternative routes to users, often without considering the impact of rerouting on future traffic conditions [8]. Research [9] found that driver route decisions depend not only on travel time information, but also on route scenery, the number of intersections, and traffic signals along the alternative route. The mentioned approach also lacks knowledge about the safety conditions of the network, which require a specific analysis based on the risk level related to road characteristics and intersection types.

The analysis of the effects of route recommendations on accident risk in urban networks [8] indicated that accident reductions, resulting from a more efficient distribution of traffic in congested networks, are small. The use of minor roads can reduce travel time, but at the same time can increase the accident frequency.

The variation of network-wide accidents caused by traffic redistribution, subject to various levels of dynamic route guidance, market penetration, and the potential of a new safety-enhanced route guidance system based on different levels and pattern simulation [10], showed approximately a 10% increase in accidents.

The available worldwide experience and research suggest the need to take into account not only the traffic performance but also an assessment of the safety performance on the alternative routes in the road networks covered by TTIS [11,12,13]. Furthermore, a reliable balance or comparison between traffic operation and road safety was never carried out for the existing TTIS for rural two-lane roads.

The aim of the paper is an assessment of the road network covered by the TTIS in terms of road safety and traffic operation, considering a dynamic traffic distribution. The paper proposes the next stage of the study of road safety on roads included in the TTIS [2] with consideration for road safety and the impact of travel time at intersections as part of the network. Therefore, for road networks included in the TTIS: 1) a detailed systematic road safety and traffic operation analysis has been done, considering the predicted number of crashes on the basis of ad hoc calibrated SPFs, 2) traffic data and their monthly variability were considered by getting data from the measuring devices implemented in the system, and 3) the relation between traffic volume and speed was developed by the authors based on empirical data from the TTIS.

## 3. Travel Time Information System for Rural Roads and Data

**Figure 1:**
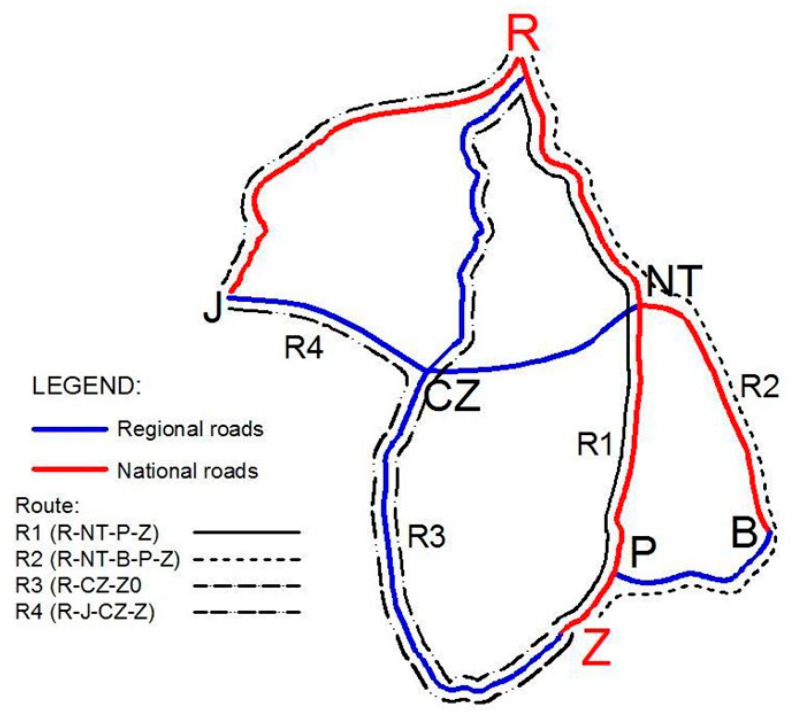
National and regional road network included in the analysis and alternative route identification.

The aim of the Travel Time Information System (TTIS) implementation was to improve traffic performance (reduction of travel time), by rerouting traffic in the road network covered by the system between two tourist sites, Zakopane (Z) and Rabka (R), in a recreational region in Poland (Figure 1). In Figure 1, the possible routes in the TTIS and the abbreviations of town names are presented.

### 3.1. Structure of the TTIS

To collect traffic data (traffic volume as well as travel and spot speed), the TTIS consists of a series of devices and sensors, i.e.,:

- Sixty Remote Traffic Microwave Sensors (RMTSs), which register traffic data (traffic volume, vehicle speed, types of vehicles) located on each route at more or less constant distances;
- Forty-four HD cameras to provide real-time control of the traffic situation, located on all routes;
- Eight Automatic Number Plate Recognition (ANPR) cameras and 16 Variable Message Signs (VMSs) to provide information to users about travel time. They are located at each intersection where a change of route is possible; and
- Ten Weather Stations (WSs) to collect weather data and provide warnings for drivers. WSs are located at the ANPR and VMS locations.

**Figure 2:**
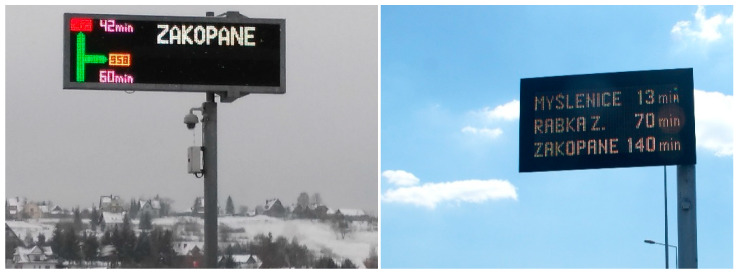
Variable message signs for travel time of the Travel Time Information System (TTIS).

The data collected by the sensors are used to estimate travel times from one location to another for different sections of roads (RMTSs) and routes (ANPR). The driver can select a route based on data on travel times provided via a VMS (Figure 2), an internet website, or a mobile app. As a result, the traffic volume distribution may vary depending on the system recommendations and the drivers’ decisions to change route between the cities of Zakopane (Z) and Rabka (R) (Figure 1).

### 3.2. Data Sample

The analyzed road network consists of national and regional two-lane rural and suburban roads, and various types of intersections (roundabouts, signalized and non-signalized intersections).

The TTIS reroutes traffic from the main route R1 (national roads R-NT-P-Z) to alternative routes (regional and national roads between Z and R, including changing points J, CZ, P, and NT) (Figure 1) characterized by various geometric standards. The road sample covered by the TTIS is made up of 156.4 km of road (including 81.5 km of regional road and 74.9 km of national road) [2].

Furthermore, the Safety Performance Functions (SPFs) for road sections and intersections were calibrated on a larger sample composed of data from two-lane roads. Those roads are located in the same region close to the routes covered by the TTIS and with similar geometric and traffic characteristics, but not affected by the system. This did not introduce any bias because there is a negligible effect of the TTIS on safety in the primary road network. This emerged from the results of the estimation of a Crash Modification Factor (CMF) which assumed a value not far from 1 [2]. That additional sample is made up of 322.9 km of road, including 184.3 km of regional road and 138.6 km of national road. This approach allows us to predict the average crash frequency for different road categories (regional and national) and intersection types (roundabouts, signalized and non-signalized intersections) comprising the network influenced by the TTIS. Table 1 and Table 2 report the summary statistics of the variables describing the sample used for the calibration of the SPFs. Roads in the system were divided into homogenous segments in terms of traffic volume (AADT), area (rural, suburban), and horizontal alignment. Intersections were categorized based on traffic control organization. Single-lane roundabouts and signalized and non-signalized four-leg intersections were distinguished. For each homogeneous segment [14,15], the segment length and the Curvature Change Rate (CCR) (Table 1) were defined as geometric covariates. For each segment and intersection, the AADT (for intersections, both the major and minor road AADT were considered) and the number of crashes, fatalities, and injuries were collected to provide the final dataset used in the SPF calibration (Table 1, while Table 2 reports the same for intersections). AADT and crash data were recorded from 2009 to 2014.

In order to assess the TTIS in terms of travel time and safety performance, the different alternative routes, consisting of sections (Table 3), were distinguished, i.e., R1 (main, the most selected route), R2, R3, and R4 (Figure 1 and Table 4). Each route is a combination of national or/and regional road sections and may be selected by the drivers between Rabka (R) and Zakopane (Z). Routes are made up of sections defined between the main intersections and can include the same segments, e.g., Route R2 (R-NT-B-P-Z) contains sections R-NT and P-Z, which are part of route R1 as well. The alternative route (for the main route R1) consisting of only regional roads is route R3.

## 4. Methodological Approach

The evaluation of the TTIS safety performance and travel time was carried out with the use of the following methodologies:

1. the calibration of SPFs for each road category and location (i.e., national/regional and rural/suburban roads) and for each intersection type (roundabouts, signalized and non-signalized intersections). This study aims to assess road safety in the entire road network included in the TTIS by juxtaposing the total predicted number of crashes for routes in various configurations of traffic distribution within the road network covered by the system; and
2. the assessment of travel time for routes included in the TTIS, based on the observed relationship between traffic volume and speed (for road sections) and delay (for intersections) with reference to traffic volume variability.

Regardless of the model calibration for segments or intersections, crashes observed at a site i in the year t (Yi,t) are typical time series data across years and can, therefore, be represented by the following simplified model structure Equation (1):

Yi,t = trend + regression term + random effects + local factors,(1)

where “trend” refers to a long-term movement due to a change in the risk factors with time, the “regression term” is of the same form as the Safety Performance Functions (SPFs), “random effects” account for latent variables across the sites, and the “local factors” refer to the dispersion between the normal safety level for similar locations and the safety level for the specific site. Random effects and local factors both contribute to the dispersion of crash counts as compared to the mean value estimated by the regression term.

The use of the Negative Binomial (NB) distribution to represent the distribution of crash counts is commonly accepted [16]. Therefore, when excluding trend effects (i.e., the phenomenon is stationary), Generalized Linear Models (GLMs) are especially useful in the context of traffic safety, for which the distribution of accident counts in a population often follows the negative binomial distribution [17,18]. In the present research work, the analysis was performed without considering possible variation in the predicted number of crashes due to the time trends because of the limited period of analysis and the target of the research work. 

Considering all this, and consistent with the state of the art in developing these models, a generalized linear modeling approach and model form was used in the elaboration, considering a negative binomial error distribution for either SPF calibrated for road sections or intersections. The important property of the GLM is the flexibility in specifying the probability distribution for the random component [19,20,21]. The model parameter estimation was performed following the maximum likelihood calibration methodology. The dispersion parameter obtained by the model calibration indicates how far the model is from a Poisson distribution, which is typically lower when a longer period is considered (lower data dispersion). Therefore, the value of the intercept is the average value in the whole period of 6 years [22,23,24].

### 4.1. Safety Performance Function Calibration for Road Segments

To compare the safety performance in terms of predicted crashes due to the changes in the Annual Average Daily Traffic (AADT) (which is the only parameter which varies due to the TTIS), ad hoc SPFs were calibrated using, as independent AADT variables, the horizontal alignment (the value of the Curvature Change Rate—CCR) and the section length on different categories of roads (national/regional) and in different locations (rural/suburban). The inclusion of other covariates, such as the segment length (L) and the horizontal alignment, helps in isolating the contribution of AADT. The inclusion of exponents for both L and AADT improves the adaptability of the model to different conditions for other variables not included in the model [25].

As a result of the previous consideration in developing SPF models, Equation (2) shows the selected model form:

E(Y) = exp(α) * AADT^β^ * L^γ^ * exp(δ*CCR), (2)

where: E(Y) is the yearly predicted number of crashes; L is the segment length [m]; AADT is the annual average daily traffic [veh./day]; and α, β, γ, and δ are regression terms.

For the regional suburban area, the variable CCR was not statistically significant and, therefore, was removed from the model.

The results of the regression analysis, obtained by using a maximum likelihood calibration methodology, are reported in Table 5. Those SPFs returned the predicted average number of crashes per year for every road section of the network based on road category and location.

**Figure 3:**
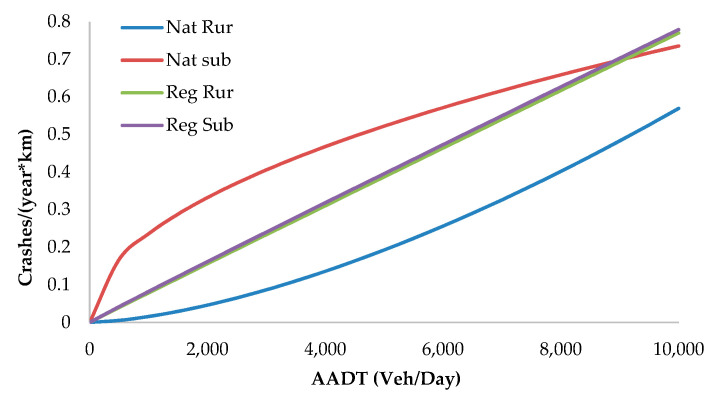
SPF diagram for segments in different road locations and road categories, with CCR equal to zero (tangent).

The best safety performances were observed on sections of national roads in rural areas (because of better geometrical standards), and the worst were in suburban areas (because of the high observed speed). Regional roads have similar safety performances in rural and suburban areas (Figure 3).

### 4.2. Safety Performance Function Calibration for Intersections

**Figure 4:**
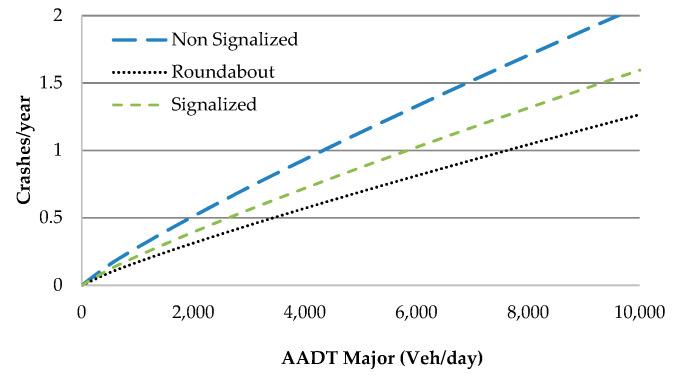
SPF diagram for different intersection types.

To estimate the predicted crash frequency for intersections, a unique SPF was calibrated using, as a categorical variable, the different intersection types, i.e., NS: non-signalized, R: roundabout, S: signalized, with a similar approach to [26]. This difference in the approach to the regression analysis between road sections and intersections was mainly due to the small sample size for each single intersection type. Only AADT was statistically significant, with a p-value lower than 0.05, and therefore it was used in the models for the major and minor roads. The model form is shown in Equation (3) and the results of calibration are shown in Table 6 and Figure 4.

E(Y) = exp(α) * AADTma^β^ * AADTmi^γ^ * exp(δi*Cat), (3)

where: E(Y) is the yearly predicted number of crashes; AADTma is the average annual daily traffic for major roads [veh./day]; AADTmi is the average annual daily traffic for minor roads [veh./day]; Cat is the categorical variable related to the type of intersection (NS: non-signalized, R: roundabout, S: signalized); α, β, and γ are regression terms of the continuous variables; and δi is the regression term of the categorical variables.

The safest intersections are the roundabouts followed by signalized intersections, while the worst performance is from non-signalized intersections, as expected (Figure 4). Therefore, the predicted crash likelihood of users traveling on alternative routes will be dependent on the type of road, the length of travel, and the number and types of intersections on the selected route.

### 4.3. Assessment of Travel Time and Variability of Traffic Volume

In order to evaluate the impact of traffic distribution on road safety, it is important to assess travel time for each route based on individual road segments and over entire networks, similar to [27]. Travel time has an impact on route selection by drivers, and as result, it affects the traffic distribution. The relationships between traffic volume and speed (for each road category and road location) were estimated by the authors.

**Figure 5:**
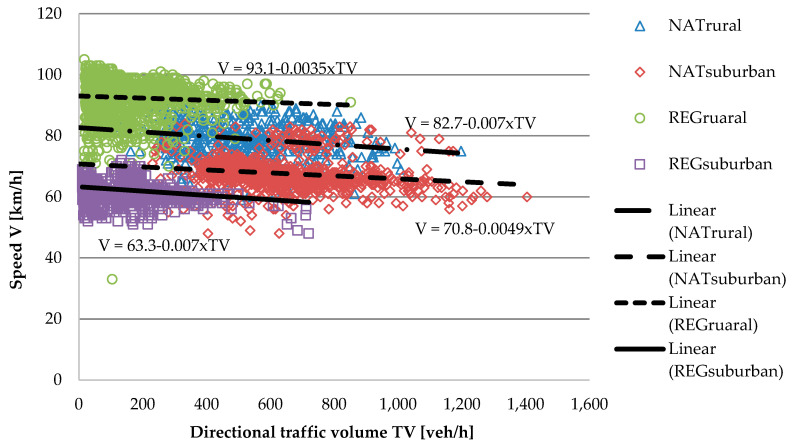
Impact of traffic volume on speed for various sections of the TTIS.

Based on empirical data, from the TTIS for each section, Figure 5 presents the relationships between speed and directional traffic volumes for national and regional roads and rural and suburban areas. In order to evaluate the traffic performance for intersections, delays as a measure of effectiveness were calculated based on the Highway Capacity Manual approach [28]. To calculate delays at the intersections, 10% of the share of peak-hour AADT was assumed. Based on travel time for sections and delays for intersections, travel time for each route was computed and compared with data from the TTIS to validate the approach.

**Figure 6:**
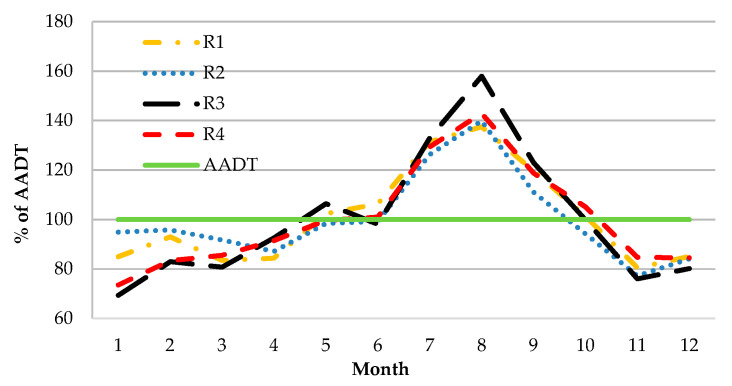
Variability of traffic volume during the year based on data from the TTIS.

In order to evaluate traffic volume variability in the TTIS, yearly traffic distributions for each route were compared (Figure 6). The rerouting of traffic during peak periods is reported in Figure 6 as it was used in Scenario 3.

The results presented in [1] confirm the need for an overall assessment of the safety performance of the system, not only for road sections, but also for intersections. The assessment of safety and traffic performance of road networks is a complex problem due to possible changes in traffic distribution at the intersections. Therefore, three different scenarios were assumed for the analysis of the impact of traffic distribution on road safety and travel time:

- an increase in AADT on main route R1 to 150% of AADT with a 10% step;
- an increase in traffic in order to balance travel time for two of the most important routes, R1 and R3; and
- an increase in traffic for all routes based on the rate from summertime (peak period).

The operation of the TTIS in terms of road safety and travel time were evaluated based on crash and traffic data.

The first scenario allows us to assess the impact of traffic volume on travel time and road safety for the main route R1, in order to show how the system operates (an increase in traffic volume with a 10% step) and indicate threshold values of traffic volume that should activate the TTIS. The second scenario allows us to assess the impact on road safety when travel time is balanced for the fastest routes, which means the system should start to work. The last scenario shows how the TTIS is working in the peak period (summertime) during traffic rerouting based on travel time.

## 5. Results and Discussion

In the present research work, three scenarios of rerouting are presented. Scenario 0 simulates the actual conditions based on observed data; an alternative scenario simulates an increase of 150% in traffic volume (Scenario 1) for the best route in terms of road standards, i.e., R1; a second alternative scenario considers the travel time of R1 equal to the route which consists of only regional roads and is the most selected alternative route for R1, i.e., R3; and the third alternative scenario considers the highest traffic volume (peak traffic) registered by the system (in August) for all routes. Those three alternative scenarios were helpful in getting values related to road safety measures or travel time and provide a basis for comparison among the different routes in different conditions.

The results of all analyzed scenarios are included in Table 7. In this table, the ratio of values for crashes and travel time as a sum of both road sections and intersections are also included. These values are calculated in relation to the main route R1 (Equation (4)).

ratio = Ri/R1,(4)

where: Ri is the value of the number of crashes or travel time for the i-th route.

A value of the ratio lower than 1 indicates that the conditions of safety or/and travel time, in comparison to the main route R1, are better.

The results indicate that for a traffic volume equal to the observed AADT (Scenario 0), route R1 has the lowest travel time (at least 41% in comparison to route R2, and even 85% of route R4), but the lowest number of crashes are predicted for route R3 (45% of crashes of R1).

**Figure 7:**
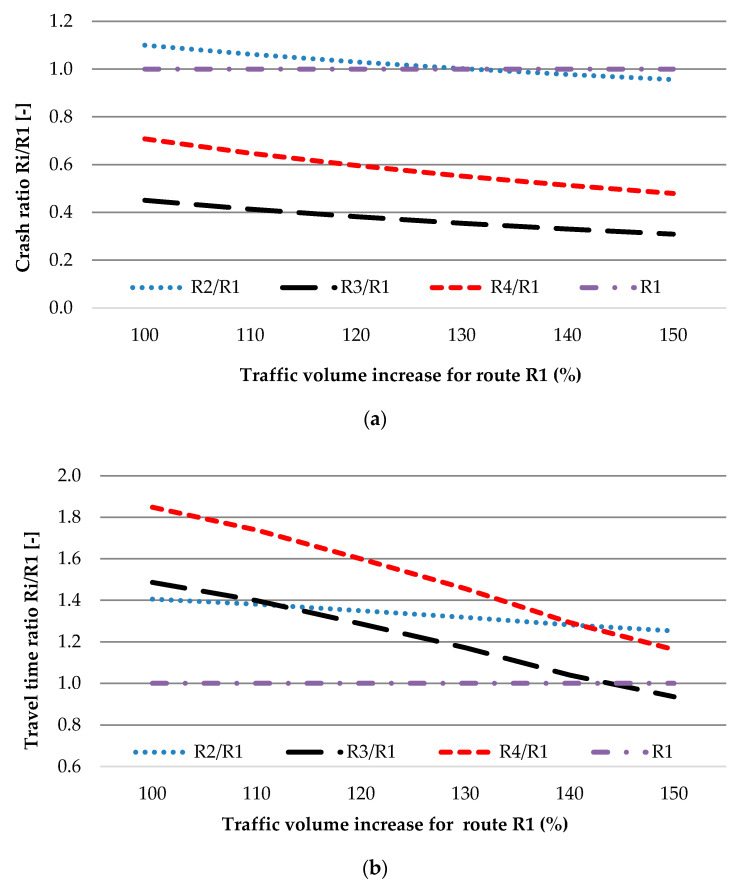
Crash (**a**) and travel time (**b**) ratio for increase in traffic volume only for route R1.

The increase in traffic only for main route R1 to 150% of AADT causes an increase in travel time and the number of crashes, as expected, taking into account the increase in risk exposure. In Figure 7, the impact of the increase in AADT for route R1 on safety and travel time by *ratio* is presented.

An increase in traffic volume equal to 143.56% of travel time is the same for routes R1 and R3 (Scenario 2). In this case, the number of crashes for routes R2, R3, and R4 is lower than for route R1. The safest route is R3, where a reduction of 88% in the predicted number of crashes, compared with main route R1, is observed.

Therefore, the safest route, R3, is very attractive, even in the condition of a high share of rerouting (Scenario 3), which can, in general, cause an increase in crashes (about 50% of crashes compared with the predicted one for route R1) during the peak period (158% of AADT). Despite the benefit to road safety, the benefits to travel time are limited to 6% (Figure 7, Scenario 2) in the case of the same increase in AADT for all routes. For the changing of traffic, as for Scenario 3, the faster route is R1. In other words, this latter condition means that the TTIS is saturated.

**Figure 8:**
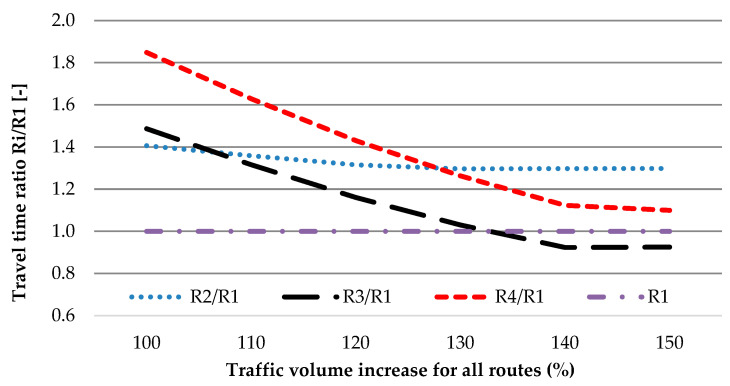
Travel time ratio for increase in traffic volume for all routes.

**Figure 9:**
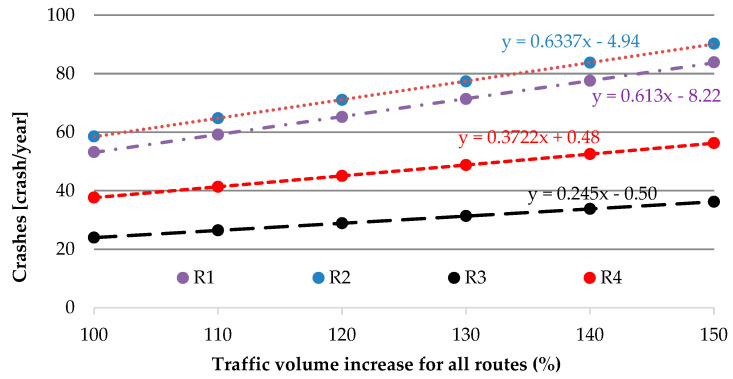
Impact of traffic volume increase for all routes on crashes.

Analysis indicates that the best alternative route (for main route R1) in the TTIS is route R3 (Figure 8; Figure 9). 

Routes R2 and R4 are not competitive compared with routes R1 and R3, both in terms of delay and safety performance. R2 is not competitive because of the greater value of travel time and predicted crash frequency in comparison to R1. It can be a good alternative route in case of local and temporary traffic interruptions on road sections belonging to other routes due to, e.g., crash occurrences or construction works. Route R4 is too long to be competitive and it is rarely used as an alternative route.

The best alternative for the main route R1 is route R3, mainly due to road safety matters. It results in a lower value of AADT (max AADT for R3 is equal to 8954 veh/day) and greater reserves of capacity. An increase in the number of crashes for R3 is lower compared with the main route R1 and is equal to 80% when considering the same percentage increase in traffic volume. In other words, it is possible to reroute 20% more vehicles to route R3 than to main route R1 to obtain the same road safety level. Therefore, it is possible to reroute more traffic in the system to R3. Travel time in peak traffic is also competitive despite the longer routes.

One of the main problems in the evaluation of the TTIS performance is related to driver choices or, in other words, how drivers use information from VMSs to select routes. Data about the variability of traffic (Figure 6) allows us to compare data on traffic distribution for one year. The peak period in the year is related to the activities of the region, whose function is mainly recreation. Based on those data (for August), the authors assume that differences in the main route R1 are the result of rerouting caused by the TTIS (higher AADT for R3 and R4). 

The same data indicate that, during wintertime (January and February), drivers prefer to use national roads (R1 and R2). It can be related to geometrical parameters (lower for regional roads) and the winter maintenance standard of roads (better for national roads).

## 6. Conclusions

The goal of the research was to develop a methodology for the evaluation of the effects on safety and travel time of the rural Travel Time Information System. For this purpose, a flexible approach was used by calibrating ad hoc SPFs for road sections (national/regional roads and rural/suburban roads), intersections, and by assessing travel time for the same possible traffic scenarios.

The presented methods, through the estimation of the influence of traffic changes in the road network with the TTIS on safety and traffic performance, allowed us to evaluate the threshold values to be used in the the TTIS’s control system. This is why, in order to efficiently operate, the TTIS has to be set with threshold values related not only to travel time but also for all the factors which can be directly influenced by the system based on the road network performance, categories, and exposure factors. Looking at the results, the use of the system makes it possible to improve the road safety that is particularly challenged when the network is made up of roads with different standards.

Therefore, given the different risks associated with different road categories, area types, intersection typologies, and the dynamic change in traffic volume (exposure factor) produced by rerouting, it is possible to estimate the TTIS effects on the road safety of the entire road network, by using the SPF models. 

The problem of the analyzed system, and common to all ITSs, is that travel time is the only factor in the decision about rerouting traffic. Displayed messages should change from the value of travel time to the recommended way of choosing from multiple criteria, which should also consider road safety.

The ITS, while providing benefits for traffic operation, changes the overall safety performance of the road network. To avoid those effects, the system management should be oriented toward more factors, finding a balance between traffic flow improvement and road safety. The methodology proposed in the paper, with a proper local calibration, can be readily used as a tool for practical applications.

Indirectly, the presented paper indicates the need to develop a navigation system with the selection of routes, taking into account the level of risk in road safety. The presented methodology can be useful to implement this approach in apps to inform drivers about risk. The changes in traffic volume observed by devices in the ITS can be used in the autocalibration procedures of SPFs. It can help to reduce the social cost of road network operation by reducing the number of crashes and victims.

## Figures and Tables

**Table 1:** Summary statistics of the dataset of road segments (Annual Average Daily Traffic—AADT—is the minimum and maximum in the whole period of analysis/CCR—Curvature Change Rate).

				Length [km]		AADT [veh/day]		CCR [deg/km]		Number of		
			No. of Road Segments	Min	Max	Min	Max	Min	Max	Crashes	Fatalities	Injured
**National roads**	**TTIS**	**suburban**	37	0.15	2.14	4892	17,564	0.0	325.7	228	19	318
		**rural**	22	0.19	2.22	6226	17,023	0.0	99.6	112	12	177
	**additional** **sample**	**suburban**	110	0.15	2.14	4892	22,022	0.0	708.5	320	41	442
		**rural**	80	0.19	2.42	4892	18,283	0.0	287.9	370	41	563
**Regional roads**	**TTIS**	**suburban**	54	0.17	2.38	2040	18,850	0.0	1095.9	145	7	190
		**rural**	19	0.12	2.16	2040	6732	0.0	449.3	62	9	104
	**additional** **sample**	**suburban**	152	0.17	2.38	2268	10,101	0.0	1044.9	249	22	312
		**rural**	76	0.13	2.43	2268	9312	0.0	796.3	151	13	249

**Table 2:** Summary statistics of the intersection dataset.

		Number of Intersections	Crashes	Fatalities	Injured
**roundabout**	**TTIS**	6	5	5	7
	**additional sample**	18	30	11	34
**signalized**	**TTIS**	3	3	2	4
	**additional sample**	7	21	1	23
**non-signalized**	**TTIS**	19	41	9	56
	**additional sample**	43	59	34	37

**Table 3:** Description of the analyzed road network covered by the TTIS by section.

(a) Sections	R-NT	NT-P	P-Z	NT-B	B-P	R-CZ	CZ-Z	R-J	J-CZ
**length [km]**	18.5	16	4.9	16.9	11.5	21.8	29.2	25.2	12.4
**min AADT [veh/day]**	14,218	14,223	17,564	9012	2040	3810	3810	7255	4281
**max AADT [veh/day]**	17,023	15,823	17,564	15,106	5891	3874	8954	7255	4751
**number of segments** **(Suburban/Rural)**	12 (7S/5R)	10 (5S/5R)	4 (4S)	13 (9S/4R)	11 (8S/3R)	17 (11S/6R)	27 (20S/7R)	26 (13S/13R)	7 (3S/4R)
**road network (National/Regional)**	N	N	N	N	R	R	R	N	R

**Table 4:** Description of the analyzed road network covered by the TTIS by route.

(b) Routes	R1 (R-NT-P-Z)	R2 (R-NT-B-P-Z)	R3 (R-CZ-Z)	R4 (R-J-CZ-Z)
**length [km]**	39.4	51.8	51.0	66.8
**min AADT [veh./day]**	14,218	5428	3810	3810
**max AADT [veh./day]**	17,564	17,564	8954	8954
**number of sections** **(Suburban/Rural)**	26 (16S/10R)	40 (28S/12R)	44 (31S/13R)	60 (36S/24R)
**road network (National/Regional)**	N	N,R	R	N,R

**Table 5:** Regression coefficient, standard error, and p-value of the Safety Perfomance Functions (SPFs) for road segments.

National Rural						
**Parameter**		**Estimate**	**Standard** Error	**Wald 95% Confidence Limits**		**Pr > ChiSq**
**Intercept**	*α*	−22.4297	2.6687	−26.56	−16.1	<0.0001
**AADT**	*β*	1.564	0.2594	1.0556	2.0725	<0.0001
**L**	γ	1.0802	0.1513	0.7836	1.3769	<0.0001
**CCR**	*δ*	0.0029	0.0015	−0.0001	0.0058	0.0495
**Dispersion parameter**		0.5404	0.139	0.2679	0.8128	--
**National suburban**						
**Intercept**	*α*	−10.3533	2.1904	−13.5478	−4.9615	<0.0001
**AADT**	*β*	0.4942	0.20089	0.0847	0.9037	0.018
**L**	γ	0.7954	0.103	0.5936	0.9973	<0.0001
**CCR**	*δ*	0.0024	0.0007	0.001	0.0038	0.0006
**Dispersion parameter**		0.4894	0.1341	0.2265	0.7523	--
**Regional rural**						
**Intercept**	*α*	−15.6614	3.3756	−21.1789	−7.9467	<0.0001
**AADT**	*β*	0.9918	0.3427	0.3201	1.6635	0.0038
**L**	γ	0.907	0.1606	0.5921	1.2218	<0.0001
**CCR**	*δ*	−0.0017	0.0009	−0.0034	0.0000	0.0411
**Dispersion parameter**		0.4855	0.1989	0.0958	0.8753	--
**Regional suburban**						
**Intercept**	*α*	−15.4732	2.0491	−18.3907	−10.3585	<0.0001
**AADT**	*β*	0.9772	0.224	0.5383	1.4162	<0.0001
**L**	γ	0.9009	0.114	0.6775	1.1243	<0.0001
**CCR**	*δ*	--	--	--	--	--
**Dispersion parameter**		0.4268	0.1357	0.1608	0.6928	--

**Table 6:** Regression coefficient, standard error, and p-value of the intersection SPFs.

Parameter		Estimate	Standard Error	Chi-Square	Pr > ChiSq
**Intercept**	*α*	−11.0055	2.694	16.69	<0.0001
**AADTma**	*β*	0.8682	0.2598	11.17	0.0008
**AADTmi**	*γ*	0.4813	0.1702	7.99	0.0089
**Non-signalized (NS)**	*δ_i_*	0.2605	0.4077	0.41	
**Roundabout (R)**	*δ_i_*	−0.2313	0.4411	0.27	
**Signalized (S)**	*δ_i_*	0	0	.	
**Dispersion**		0.6943	0.2181		

**Table 7:** Values of crashes and travel time for all routes (the lowest values are in bold).

	Route			*Ratio*			
	R1	R2	R3	R4	R2/R1	R3/R1	R4/R1
	Observed AADT (scenario 0)						
**Crashes (SPF) [crash/year]**	53.23	58.54	**23.99**	37.68	1.10	0.45	0.71
**Crash rate** [Crash*10^6^/(365*AADT*km)]	0.17	0.16	0.13	**0.10**	-	=	-
**Travel time + delay [min]**	**33.01**	46.41	49.07	61.02	1.41	1.49	1.85
	Increase in traffic volume to 150% of AADT for R1 (scenario 1)						
**Crashes (SPF) [crash/year]**	80.41	76.90	**24.87**	38.56	0.96	0.31	0.48
**travel time + delay [min]**	52.48	66.12	**49.07**	61.02	1.25	0.94	1.16
	Increase in traffic volume to 143.56% of AADT for R1 (scenario 2) – the same travel time for R1 ad R3						
**Crashes (SPF) [crash/year]**	76.75	74.43	**24.76**	38.45	0.97	0.32	0.50
**Travel time + delay [min]**	**49.07**	62.33	**49.07**	61.02	1.27	1.00	1.24
	Increase in traffic volume to value of peak period (in August) for all routes (scenario 3) – (R1 = 137.5%. R2 = 140%. R3 = 158%. R4 = 143%)						
**Crashes (SPF) [crash/year]**	76.29	82.94	**37.08**	55.30	1.09	0.49	0.72
**Travel time + delay [min]**	**57.57**	75.09	63.00	74.10	1.30	1.09	1.29
